# Ecological Drivers of Shark Distributions along a Tropical Coastline

**DOI:** 10.1371/journal.pone.0121346

**Published:** 2015-04-08

**Authors:** Peter M. Yates, Michelle R. Heupel, Andrew J. Tobin, Colin A. Simpfendorfer

**Affiliations:** 1 Centre for Sustainable Tropical Fisheries and Aquaculture and College of Marine and Environmental Sciences, James Cook University, Townsville, Queensland, Australia; 2 Australian Institute of Marine Science, Townsville, Queensland, Australia; University of California Davis, UNITED STATES

## Abstract

As coastal species experience increasing anthropogenic pressures there is a growing need to characterise the ecological drivers of their abundance and habitat use, and understand how they may respond to changes in their environment. Accordingly, fishery-independent surveys were undertaken to investigate shark abundance along approximately 400 km of the tropical east coast of Australia. Generalised linear models were used to identify ecological drivers of the abundance of immature blacktip *Carcharhinus tilstoni*/*Carcharhinus limbatus*, pigeye *Carcharhinus amboinensis*, and scalloped hammerhead *Sphyrna lewini* sharks. Results indicated general and species-specific patterns in abundance that were characterised by a range of abiotic and biotic variables. Relationships with turbidity and salinity were similar across multiple species, highlighting the importance of these variables in the functioning of communal shark nurseries. In particular, turbid environments were especially important for all species at typical oceanic salinities. Mangrove proximity, depth, and water temperature were also important; however, their influence varied between species. Ecological drivers may promote spatial diversity in habitat use along environmentally heterogeneous coastlines and may therefore have important implications for population resilience.

## Introduction

Understanding the relationship between sharks and their environment can facilitate the identification of critical habitats for the sustainable management and conservation of shark populations [1]. In particular, the factors that influence use of nursery areas by young sharks have been a focus of recent research [2, 3]. Shark nurseries are defined as areas with (1) relatively high abundance of young sharks, (2) site fidelity and (3) stability in use across multiple years [4]. The use of nursery areas presumably enhances the fitness of young sharks [5], which in turn can influence population productivity [6]. Therefore, data on the location and functioning of shark nurseries may enhance management and conservation of shark populations.

Coastal environments can provide young sharks with abundant prey [7, 8] and refuge from larger-bodied predators [9]. In addition, some coastal regions are used by multiple species and may function as communal shark nurseries [10]. The distribution and habitat use of sharks in coastal environments can often be attributed to spatio-temporal variation in environmental conditions [11]. Coastal environments can be susceptible to a range of human impacts (reviewed in [12]) and environmental change [13], and the identification of factors that influence the habitat use of coastal sharks can improve understanding of how they may respond to changes within their environment.

A wide range of abiotic variables are thought to influence the habitat use of coastal sharks (reviewed in [11]). For example, water temperature [14], depth [15], salinity [3, 16], turbidity [17] and dissolved oxygen concentration (DO; [18, 19]) have been identified as important factors for multiple species. Influences of abiotic variables may be dictated by a species’ physiological requirements [11]. For example, ectothermic sharks are hypothesised to use behavioural thermoregulation to optimise energetic uptake and expenditure (reviewed in [20]), and avoid lethal temperatures [21]. Coastal sharks may also occupy particular salinities to reduce the metabolic demands of osmoregulation [3], although their salinity preferences can change with age [22–24]. Thus a range of factors, including physiology, age and other biotic variables are likely to shape how abiotic conditions influence a species.

Biotic variables are widely cited as determinants of habitat selection by sharks [25–27]. For example, predation risk was implicated in the habitat use and aggregation of juvenile lemon sharks *Negaprion brevirostris* within a subtropical mangrove-inlet [28]. Shark abundance has also been linked with the abundance of potential teleost prey, albeit over broad spatial scales [26]. In addition, biotic ecosystem features such as mangroves and seagrass beds may provide multiple benefits including abundant prey and refuge from predators [29, 30]. Relationships between sympatric young sharks, and other life-history stages, may also influence habitat use. Competition for limited resources may necessitate inter- or intraspecific partitioning of space and food resources [29, 31]. Conversely, aggregation behaviour may reduce susceptibility to predation. For example, schooling fish benefit from the dilution of predation risk [32], and therefore young sharks may derive similar benefits by having similar spatio-temporal occurrences and habitat use patterns [33–35]. A variety of biotic factors are important for the habitat use of coastal sharks including varied trade-offs between predation risk and energetic requirements.

Although a large portion of research on shark nurseries has occurred across restricted spatial scales (e.g. [9, 36]), there are examples of intraspecific variation in habitat use between nearby inshore systems. For example, contrasting habitat use patterns of immature sandbar sharks *Carcharhinus plumbeus* between two adjacent bays along the eastern United States may have coincided with spatial variation in hydrodynamics and predation pressure [37, 38]. Salinity was most influential in the habitat use of juvenile bull sharks in the Caloosahatchee River, Florida [23], but exerted a relatively small influence relative to DO in the Florida Everglades [18]. Salinity fluctuations were larger in the Caloosahatchee River, which may have necessitated a more pronounced salinity response by sharks occurring there compared to those in the Florida Everglades [18]. Intraspecific variations in habitat use highlight the importance of sampling within multiple habitats to gain a more comprehensive understanding of shark habitat use across a region.

Coastal habitats along north-eastern Australia support a diverse and abundant shark assemblage [39], within which immature Australian blacktip *Carcharhinus tilstoni*, common blacktip *Carcharhinus limbatus*, pigeye *Carcharhinus amboinensis*, and scalloped hammerhead *Sphyrna lewini* sharks are relatively abundant [40]. The scarcity of data on the distribution and abundance of these species hinders the identification of critical habitats and understanding of the impacts of environmental change. The aim of this study was to investigate the influence of abiotic and biotic variables on the distribution of immature blacktip, pigeye and scalloped hammerhead sharks across a broad spatial scale and provide information for the sustainable management of important habitats.

## Methods

### Ethics statement

Sampling was conducted under a Queensland Department of Agriculture, Fisheries and Forestry General Fisheries Permit (no. 144482) and Great Barrier Reef Marine Park Authority Permit (no. G11/34618.1). All procedures were approved by James Cook University’s Animal Ethics Committee (no. A1566, 1933).

### Study area

**Fig 1:**
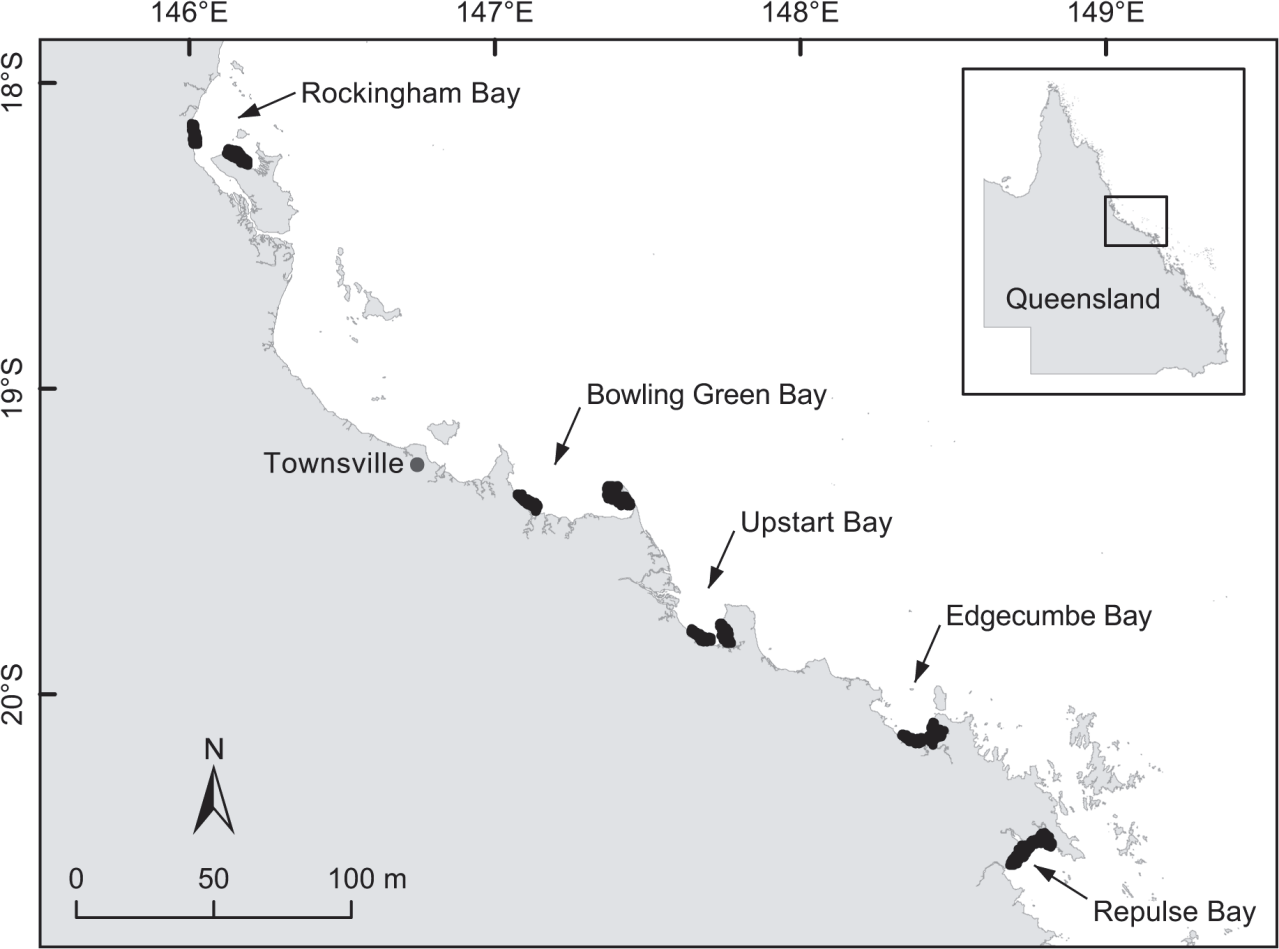
Study area. Spatial distribution of longline and gill-net sampling (indicated in black) within the five study bays along tropical north Queensland.

This study was conducted within five bays spanning c. 400 km of the tropical north Queensland coastline (Fig. 1): Rockingham, Bowling Green, Upstart, Edgecumbe and Repulse Bays. The bays are shallow (predominantly < 15 m depth) and sheltered from ocean swells by the Great Barrier Reef. As a result, bays were dominated by silty substrates with mudflat or mangrove-lined foreshores. Environmental conditions across the study region were spatially and temporally variable. For example, mangrove extent ranged from c. 29 km^2^ in Edgecumbe Bay up to c. 205 km^2^ in Rockingham Bay. The supply of freshwater from rivers typically varied depending on catchment size and the spatial distribution of rainfall [41]. In addition, rainfall was highly seasonal with 60–80% typically occurring during the summer wet season (November–April; [41]).

### Field methods

Two methods were used to sample across a broad range of shark sizes. During a total of 162 days of sampling, 453 longline shots and 343 gill-net shots were deployed totalling 370.1 and 309.8 hours, respectively (Table 1). Longline and gill-net sampling was conducted in water depths ranging from 0.5 to 5 m. Bottom-set gill-nets, up to 400 m length and comprised of 11-cm-stretched mesh, were deployed for c. 1 h and checked every 15 min to facilitate tagging and release. Bottom-set longlines were comprised of 800 m of 6-mm nylon mainline with an anchor and float at both ends. Gangions were attached to the mainline c. 8–10 m apart; and were comprised of 1 m of 4-mm nylon cord, 1 m of 1.5-mm wire leader, and a baited size 14/0 Mustad tuna circle hook. A variety of fresh and frozen baits were used, which consistently comprised a combination of squid *Loligo sp*. and various teleost fish (butterfly bream *Nemipterus sp*., blue threadfin *Eleutheronema tetradactylum* and mullet *Mugil cephalus*). Up to two longlines were deployed simultaneously for 40–90 min sets.

**Table 1:** Summary of fishing effort and proportion of area/coastline sampled in five study bays.

	Longline				Gill-net				Proportion of bay area sampled	Proportion of coastline sampled
	Number of shots	Total soak hours	Standardised soak hours	Hooks per shot	Number of shots	Total soak hours	Standardised soak hours	Net lengths(m)		
Rockingham	93	74.3	40.3	54 ± 9	67	58.0	134.2	100, 200, 400	0.08	0.24
Bowling Green	91	76.3	39.6	52 ± 11	66	64.6	181.5	100, 200, 400	0.13	0.31
Upstart	93	75.4	39.7	52 ± 9	76	63.1	121.4	100, 200	0.15	0.38
Edgecumbe	88	72.9	39.3	54 ± 11	69	64.7	170.9	100, 200, 400	0.12	0.35
Repulse	88	71.2	38.0	53 ± 9	65	59.4	174.9	100, 200, 400	0.19	0.41
Total	453	370.1	196.9	53 ± 10	343	309.8	782.9	100, 200, 400	-	-

Data are from years 2012 - 2014 combined. Standardised soak hours = sum of 100-hook-hours and 100 m-net-hours for longlines and gill-nets, respectively. Hooks per shot refers to mean number of hooks ± SD. Bay-area and coastline coverage were the same for both sampling methods.

Between January 2012 and March 2014, eight rounds of fishery-independent surveys were undertaken to collect data on the shark fauna in each of the bays. Within each bay, sampling was randomised within sixteen 0.9-km-wide transects. Each bay was sampled over four days allowing for a minimum of eight gill-net samples bay^–1^ round^–1^ and 10 longline samples bay^–1^ round^–1^.

Captured sharks were identified to species level, tagged on the first dorsal fin (Rototag or Superflex tag; Dalton, Oxfordshire, UK), measured, sexed, assessed for clasper calcification, examined for umbilical scar condition, and released at their capture site. Stretch total length (STL) was determined according to Compagno [22]. Small sharks (≤ 1 m) were placed ventral side down on a measuring board and measured to the nearest mm with the upper lobe of the caudal fin depressed in line with the body axis. Larger sharks were secured beside the boat and measured to the nearest cm using a measuring tape. Additional measurements of fork length and pre-caudal length were recorded.

Life-history stage was determined using length-at-age data (reviewed in [42, 43]), observation of umbilical scars which indicated recent birth, and clasper calcification which indicated sexual maturity in males. Two morphologically similar species, Australian blacktip and common blacktip, were indistinguishable in the field and therefore grouped together as unidentified blacktip sharks. The length-at-age estimates for the Australian blacktip were used to determine life-history stage to ensure that no mature sharks were misclassified as immature (similar to [39, 40]).

For each fishing deployment, water depth was recorded to the nearest 0.1 m using the vessel’s depth sounder (Garmin Echo 500C) and taken as the mean of measurements from both ends of the deployment. Sea-surface water temperature (°C), salinity (ppt), and DO (mg/L) were recorded using a YSI Model 85 multiprobe (YSI Incorporated). Secchi depth was recorded to the nearest 0.1 m as a proxy for turbidity. The secchi disk was visible on the sea floor during 7% of fishing shots, however these occurrences were spread across the full range of depths sampled. Therefore, the secchi disk being visible on the bottom was not a reliable indicator of low turbidity and so these occurrences were treated as missing values (e.g. in shallow water the secchi disk may be visible on the bottom even in relatively turbid conditions). Geographic coordinates were recorded at both ends of fishing deployments. Mangrove proximity was calculated using ArcMap 10.2.1 (ESRI) as the shortest straight-line distance to any mangrove polygon (km) within the same bay.

## Data Analysis

### Site variability

To investigate broad-scale environmental heterogeneity, Kruskal-Wallis rank sum tests (R package *stats*; [44]) were used to identify significant variations in environmental measurements between bays. Significant variations were investigated with a multiple comparison test which identified where variations existed (R package *pgirmess*; [45]).

### Variables influencing shark catch

Generalised linear models (R package *MASS*; [46]) were used to examine the relationship between environmental variables and the abundance of blacktip, pigeye and scalloped hammerhead sharks across the study region. Longline and gill-net data were analysed separately. The abundance of immature sharks within putative nursery areas was the focus of this study therefore analyses were limited to immature individuals. Low abundance of scalloped hammerhead sharks in longline samples precluded further analysis of this sampling method for this species. Prior to model fitting, data exploration was carried out according to Zuur et al. [47] and Zuur et al. [48]. Cleveland dotplots were used to check for outliers. Conditional boxplots, pairwise scatterplots, Pearson correlation coefficients and variance inflation factors (VIF, R package *car*; [49]) were used to investigate relationships between variables. Spatio-temporal variables (bay and sampling round) were confounded with multiple environmental variables and were therefore excluded from analyses. In addition, high VIF (i.e. ≥ 3; [50]) indicated collinearity between temperature and DO. Ninety-seven percent of DO measurements were > 4.5 mg/L thus DO was deemed unlikely to be a limiting factor for sharks and was excluded from further analyses. Subsequently, VIF were < 1.3 for remaining variables (Table 2). There were only two cases of a shark being captured twice during the same four-day trip therefore we assumed independence of individual fishing samples.

**Table 2:** Spatial variation in ecological variables.

	Depth(m)	Temperature (°C)	Salinity (ppt)	Secchi depth/turbidity(m)	Distance to mangroves (km)
Rockingham	2.7 ± 1.0^a^	27.1 ± 2.6^a^	32.3 ± 4.0^b^	1.0 ± 0.5^a^	0.95 ± 0.65^a^
Bowling Green	2.9 ± 1.2^a^	26.2 ± 3.0^ab^	34.9 ± 1.4^a^	1.2 ± 0.7^ab^	2.34 ± 1.24^b^
Upstart	2.8 ± 1.1^a^	25.6 ± 3.6^b^	35.1 ± 1.2^a^	1.7 ± 0.8^c^	0.85 ± 0.46^a^
Edgecumbe	2.8 ± 1.1^a^	25.3 ± 3.5^b^	34.8 ± 1.3^a^	2.0 ± 0.7^d^	0.91 ± 0.54^a^
Repulse	2.8 ± 1.0^a^	25.8 ± 3.8^b^	32.4 ± 3.7^b^	1.4 ± 0.8^b^	1.59 ± 1.01^c^
Overall	2.8 ± 1.1	26.0 ± 3.4	33.9 ± 2.9	1.4 ± 0.8	1.32 ± 1.01

Data were pooled across sampling rounds and years. Values are mean ± 1 SD. For each variable, bays without a shared letter were significantly different (multiple comparison test following Kruskal-Wallis rank sum test, *df* = 4, *P* < 0.05).

To avoid over-fitting of the data with spurious relationships, investigations were limited to an *a priori* selection of ecologically relevant covariates and interactions [51, 52]. For each species/sampling-method combination, the following ‘starting’ model was created containing the main effects and interactions of interest:

$$\begin{array}{l} \text{log(}Abundance_{i})=\beta_{1}+\beta_{2}\times Depth_{i}+\beta_{3}\times Temperature_{i}+\beta_{4}\times Salinity_{i}+\beta_{5}\times Secchi\;depth_{i}+\beta_{6}\times Mangrove\;distance_{i}+\\ \beta_{7}\times Secchi\;depth_{i}*Depth_{i}+\beta_{8}\times Secchi\;depth_{i}*Salinity_{i}+offset\text{(}log(Fishing\;effort_{i}\text{))} \end{array}$$

Shark abundance was assumed to be negative binomial distributed, and the logarithm link between expected shark abundance (*Abundance*_*i*_) and the selected covariates ensured that all fitted values were non-negative. Standardised fishing effort was calculated as the logarithm of 100-hook hours for longlines or the logarithm of 100m-net hours for gill-nets. Interaction terms were selected based on putative implications of depth and turbidity for vulnerability to predation [5, 25], and the potential for interaction between turbidity and salinity during the summer wet season [53].

To identify the most influential drivers of shark abundance, a dredge function (R package *MuMIn*; [54]) was used to identify more-parsimonious nested models according to the Akaike Information Criterion (AIC). This approach required prior omission of samples containing missing values (31 longline shots and 27 gill-net shots). The rule of marginality was applied whereby interactions were only considered in models that contained both main effects. Additionally, a maximum of eight parameters were specified per model and the offset variable was ‘fixed’ within all models. A ‘confidence set’ of models with ΔAIC < 2 were considered equivalent and included in model averaging; from which the Relative Variable Importance values (RVI; calculated from the sum of AIC weights of models within the confidence set in which the parameter of interest appears) were used to identify important variables. If multiple variables shared the same RVI, the magnitude of the standardised model-averaged coefficient provided an alternative measure of relative influence. A single model containing only highly influential variables, identified as those preceding a sharp decline in RVI, was used for visual representation of variable effects (R package *visreg*; [55, 56]), calculation of explained deviance, and assessment of adherence to model assumptions. Cook’s distances were used to check for observations with disproportionally high influence. Pearson residuals were plotted against fitted shark abundance as well as included and excluded covariates to check for homogeneity, independence and model fit. Pearson residuals were plotted by geographic position according to their sign and magnitude. Minor spatial structuring of residuals was observed for pigeye and scalloped hammerhead sharks, however this was not improved by the inclusion of bay, transect group or sampling round as random intercepts (R packages *glmmADMB* and *lme4*; [57, 58]).

## Results

### Catch composition

**Fig 2:**
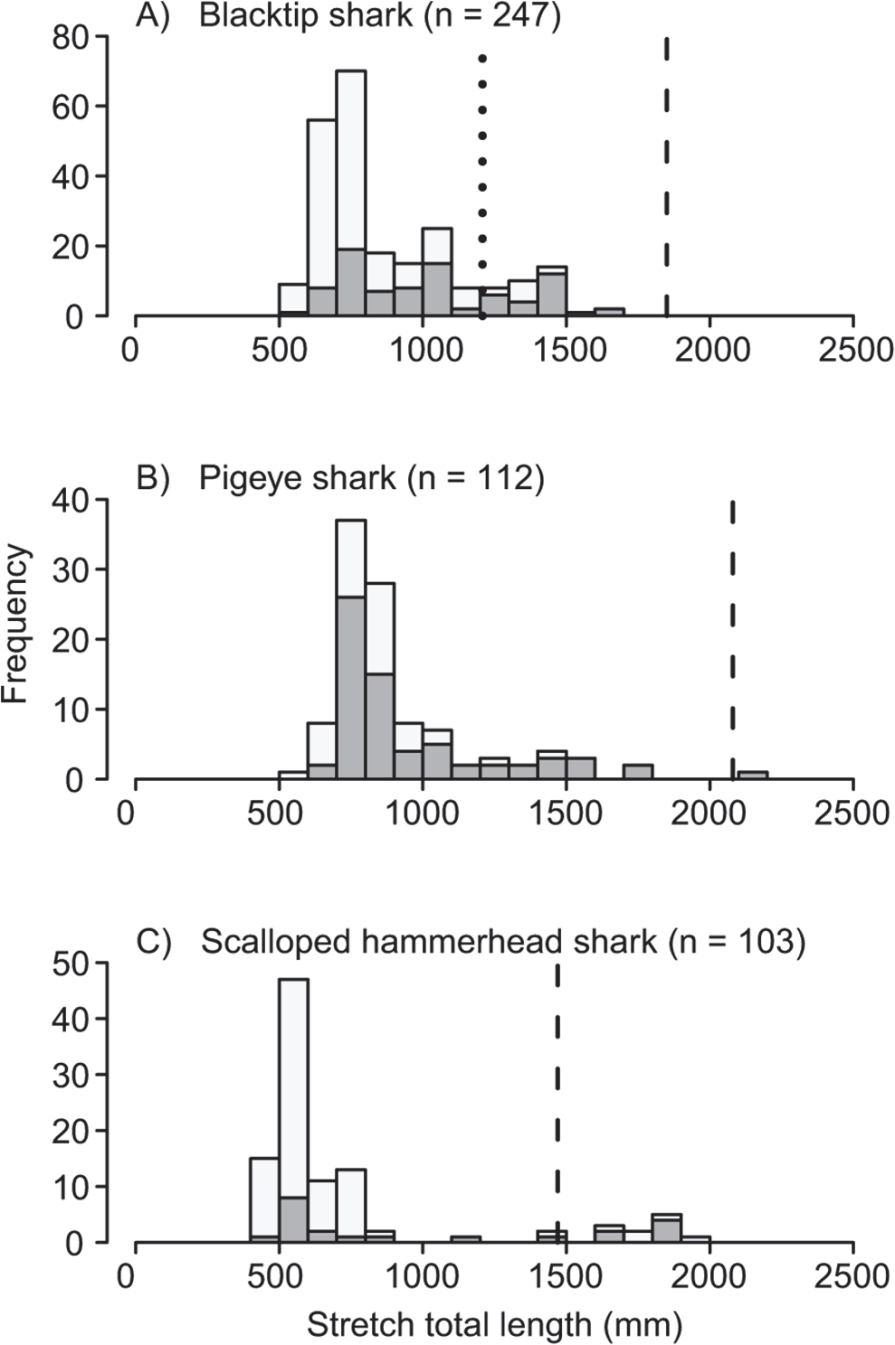
Length-frequency distributions of blacktip, pigeye and scalloped hammerhead sharks. Bar shading denotes the sampling method (dark grey = longline; light grey = gill-net). Lengths at 50% maturity (reviewed in [42, 43]) are denoted by broken lines (larger dashes for the common blacktip shark; A). The lengths at 50% maturity for common blacktip, pigeye and scalloped hammerhead sharks differ between sexes and so the smallest is given (male in all cases).

A total of 1987 sharks were captured from six families. Of the 22 species encountered, carcharhiniform sharks made up 99.2% of the total catch. The catch of immature sharks was dominated by blacktip (31%), pigeye (17%) and scalloped hammerhead (14%) sharks. Length-frequency histograms indicated that these species were predominantly immature (Fig. 2).

### Site variability

There was significant variation in water temperature (Kruskal-Wallis rank sum test; *χ* ^*2*^ = 23.7, *df* = 4, *P* < 0.001), salinity (*χ* ^*2*^ = 142.4, *df* = 4, *P* < 0.001), turbidity (*χ* ^*2*^ = 167.4, *df* = 4, *P* < 0.001), and mangrove proximity (*χ* ^*2*^ = 187.7, *df* = 4, *P* < 0.001) between study bays (Table 2). For example, mean salinity was ≥ 2.4 ppt lower in Rockingham and Repulse Bays compared to the other three bays (Table 2). In addition, turbidity was significantly lower (i.e. secchi depth was higher) in Edgecumbe Bay, followed by Upstart Bay. This spatial heterogeneity created an ideal study region for investigating the drivers of shark abundance. Sampled water depths did not vary significantly between bays (Kruskal-Wallis rank sum test; *χ* ^*2*^ = 1.1, *df* = 4, *P* = 0.90), confirming that a comparable spectrum of depths were sampled across bays.

### Variables influencing shark catch

**Fig 3:**
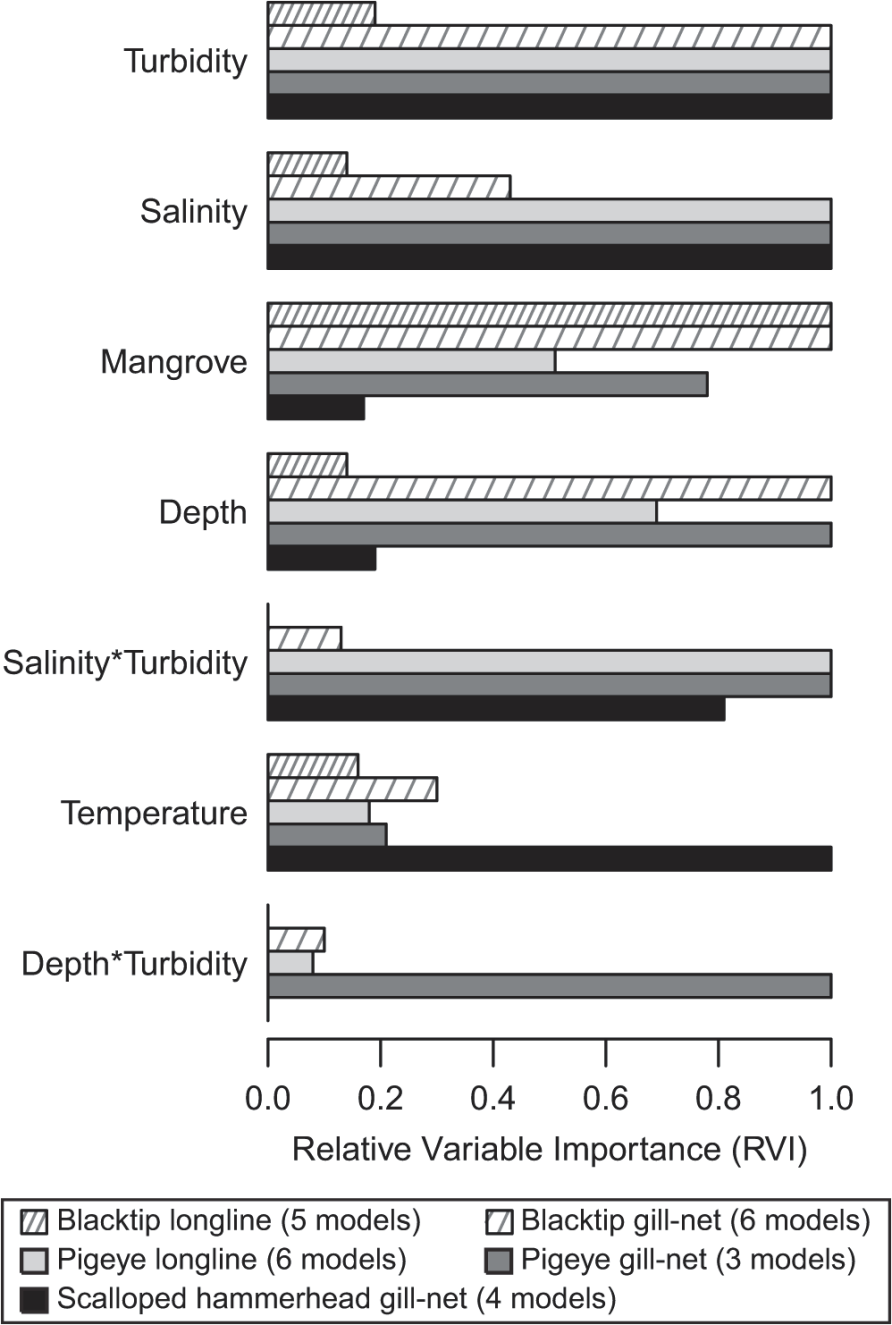
Relative importance of ecological variables. Parameters are listed according to mean Relative Variable Importance (RVI) across all species/sampling-method combinations. Parameters that were ubiquitous within the confidence set (i.e. models with ΔAIC < 2) have a RVI value of 1.0. The numbers of models included in model averaging are provided in parentheses.

Variation in shark abundance was associated with complex combinations of main effects and interactions. Overall, turbidity and salinity were the most influential variables on shark abundance (Fig. 3). Most notably, excluding blacktip sharks on longlines, turbidity was present in all best-performing models (i.e. those with ΔAIC < 2; Table 3). Mangrove proximity, depth and water temperature were also important however their influence varied between species.

**Table 3:** Comparison of best-performing models of immature shark abundance. Each row contains the intercept and coefficients that comprised a single model, along with the number of parameters (df), log-likelihood and Akaike metrics.

Intercept	Turbidity	Salinity	Mangrove	Depth	Temperature	Salinity*Turbidity	Depth*Turbidity	*df*	Log-likelihood	AIC	ΔAIC	*w*
Blacktip shark *Carcharhinus tilstoni/ C*. *limbatus* on longlines												
-0.60	-	-	-0.47	-	-	-	-	3	-168.5	343.0	0.00	0.266
-0.34	-0.19	-	-0.46	-	-	-	-	4	-168.2	344.4	1.34	0.136
0.03	-	-	-0.47	-	-0.02	-	-	4	-168.4	344.8	1.74	0.111
-0.71	-	-	-0.49	0.04	-	-	-	4	-168.5	345.0	1.92	0.102
Blacktip shark *Carcharhinus tilstoni/ C*. *limbatus* in gill-nets												
-0.59	-1.28	-	-0.31	0.30	-	-	-	5	-237.7	485.5	0.00	0.203
-2.48	-1.35	0.06	-0.32	0.32	-	-	-	6	-237.1	486.3	0.80	0.136
-1.92	-1.21	-	-0.29	0.27	0.05	-	-	6	-237.2	486.3	0.85	0.133
-5.56	2.83	0.15	-0.33	0.32	-	-0.12	-	7	-236.5	487.0	1.50	0.096
-3.59	-1.28	0.05	-0.30	0.29	0.05	-	-	7	-236.6	487.3	1.80	0.083
-0.53	-1.34	-	-0.31	0.28	-	-	0.02	6	-237.7	487.5	1.99	0.075
Pigeye shark *Carcharhinus amboinensis* on longlines												
-15.07	14.36	0.43	-0.31	0.36	-	-0.45	-	7	-157.1	328.1	0.00	0.182
-14.46	13.89	0.40	-	0.29	-	-0.44	-	6	-158.1	328.2	0.07	0.176
-13.22	14.19	0.39	-	-	-	-0.44	-	5	-159.2	328.4	0.25	0.160
-13.46	14.59	0.41	-0.22	-	-	-0.46	-	6	-158.7	329.3	1.17	0.101
-13.73	13.87	0.42	-0.33	0.38	-0.04	-0.44	-	8	-156.9	329.8	1.64	0.080
-13.52	13.54	0.39	-	0.31	-0.02	-0.43	-	7	-158.0	330.0	1.89	0.071
Pigeye shark *Carcharhinus amboinensis* in gill-nets												
-27.88	30.01	0.53	-0.79	3.04	-	-0.77	-1.71	8	-80.7	177.4	0.00	0.432
-27.21	30.52	0.49	-	3.03	-	-0.78	-1.86	7	-82.7	179.3	1.90	0.167
-27.29	29.92	0.52	-0.80	3.05	-0.01	-0.77	-1.71	9	-80.7	179.4	1.98	0.160
Scalloped hammerhead shark *Sphyrna lewini* in gill-nets												
-7.64	8.20	0.08	-	-	0.15	-0.29	-	6	-131.8	275.6	0.00	0.287
-8.04	8.43	0.09	-	0.15	0.14	-0.30	-	7	-131.7	277.3	1.66	0.125
-0.99	-1.37	-0.14	-	-	0.17	-	-	5	-133.7	277.3	1.70	0.123
-7.65	8.24	0.09	-0.06	-	0.15	-0.29	-	7	-131.8	277.6	1.92	0.110

All models contained fishing effort as an offset variable. AIC = Akaike Information Criterion, ΔAIC = increase in AIC relative to the lowest-AIC model, *w* = Akaike weight.

#### Blacktip shark

**Fig 4:**
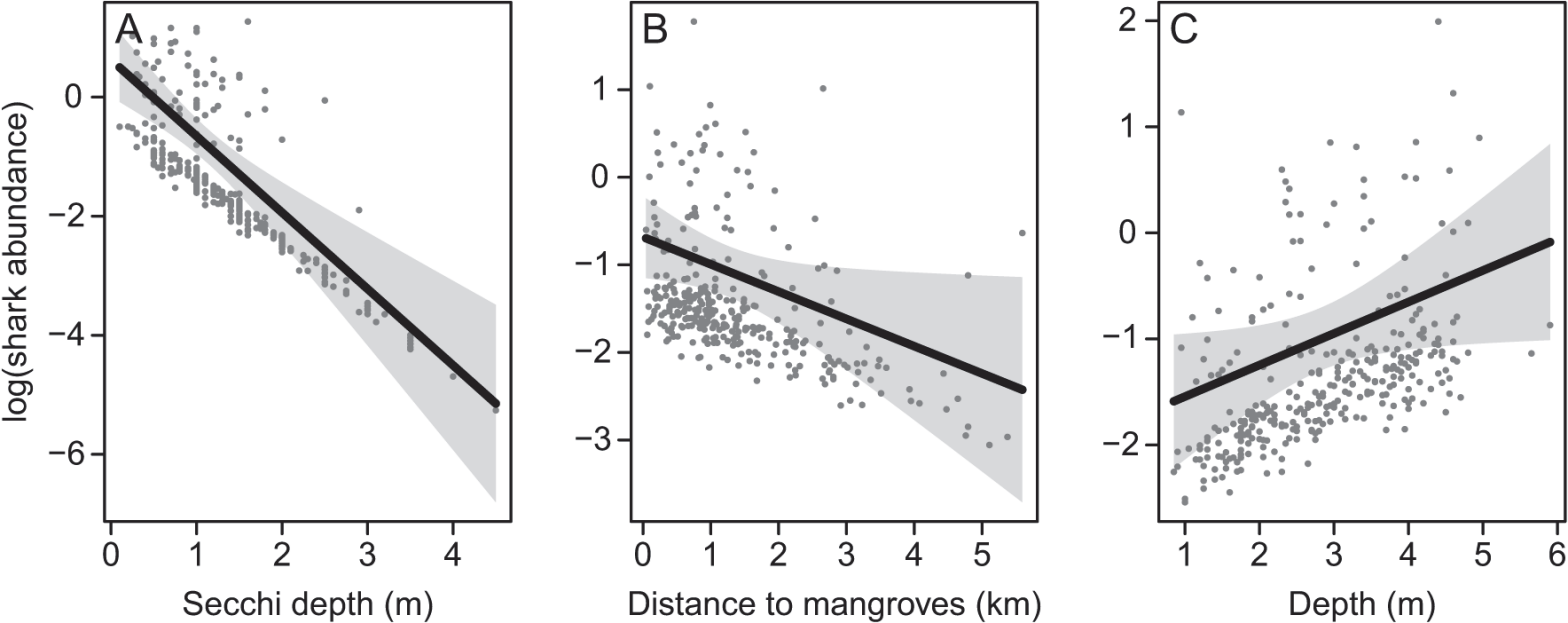
Modelled relationships between the abundance of immature blacktip sharks in gill-nets and highly influential variables. Shading represents 95% confidence intervals and points are partial residuals. Effects were plotted with additional variables held at their medians. The model containing turbidity, distance to mangroves and depth had dispersion statistic = 1.15 and negative binomial variance parameter *k* = 0.32. Note that low values of secchi depth indicate high turbidity.

A total of 86 and 161 blacktip sharks were captured using longlines and gill-nets, respectively. Of these, 60 and 141 immature individuals were included in longline and gill-net analyses, respectively. For longlines, a weakly significant effect of mangrove proximity was detected (Table 4), however the explained deviance of 4% indicated that the influence of this variable was negligible. Turbidity and depth were highly influential in gill-net samples (Fig. 4; Table 4). In addition, the influence of mangrove proximity in gill-net samples corroborated the otherwise equivocal longline results. Overall, blacktip shark abundance decreased with decreasing turbidity (i.e. increasing secchi depth) and distance from mangroves, and increased with depth (Fig. 4). These three variables were present in all best-performing models (Table 3) and together explained 18% of deviance in blacktip shark abundance in gill-nets.

**Table 4:** Effects of highly influential variables (identified using Relative Variable Importance values, RVI). Standardised model-averaged coefficients (with shrinkage) ± standard error are followed by the associated *P*-value in parentheses (i.e. Pr(>|Z|)).

	*C*. *tilstoni/ C*. *limbatus*		*C*. *amboinensis*		*S*. *lewini*
	Longline	Gill-net	Longline	Gill-net	Gill-net
Turbidity		-0.48 ± 1.28 (0.71)**	17.42 ± 3.97 (< 0.001)	36.81 ± 11.52 (0.001)	5.53 ± 5.30 (0.30)
Salinity			1.87 ± 0.67 (0.006)	2.35 ± 1.27 (0.07)	0.14 ± 0.47 (0.77)
Mangrove	-0.93 ± 0.41 (0.02)	-0.27 ± 0.13 (0.04)*			
Depth		0.26 ± 0.15 (0.08)*		5.12 ± 1.32 (< 0.001)	
Salinity*Turbidity			-19.14 ± 4.20 (< 0.001)	-33.29 ± 11.44 (0.004)	-7.11 ± 5.61 (0.21)
Temperature					0.55 ± 0.28 (0.05)
Depth*Turbidity				-8.87 ± 3.16 (0.005)	

**Fig 5:**
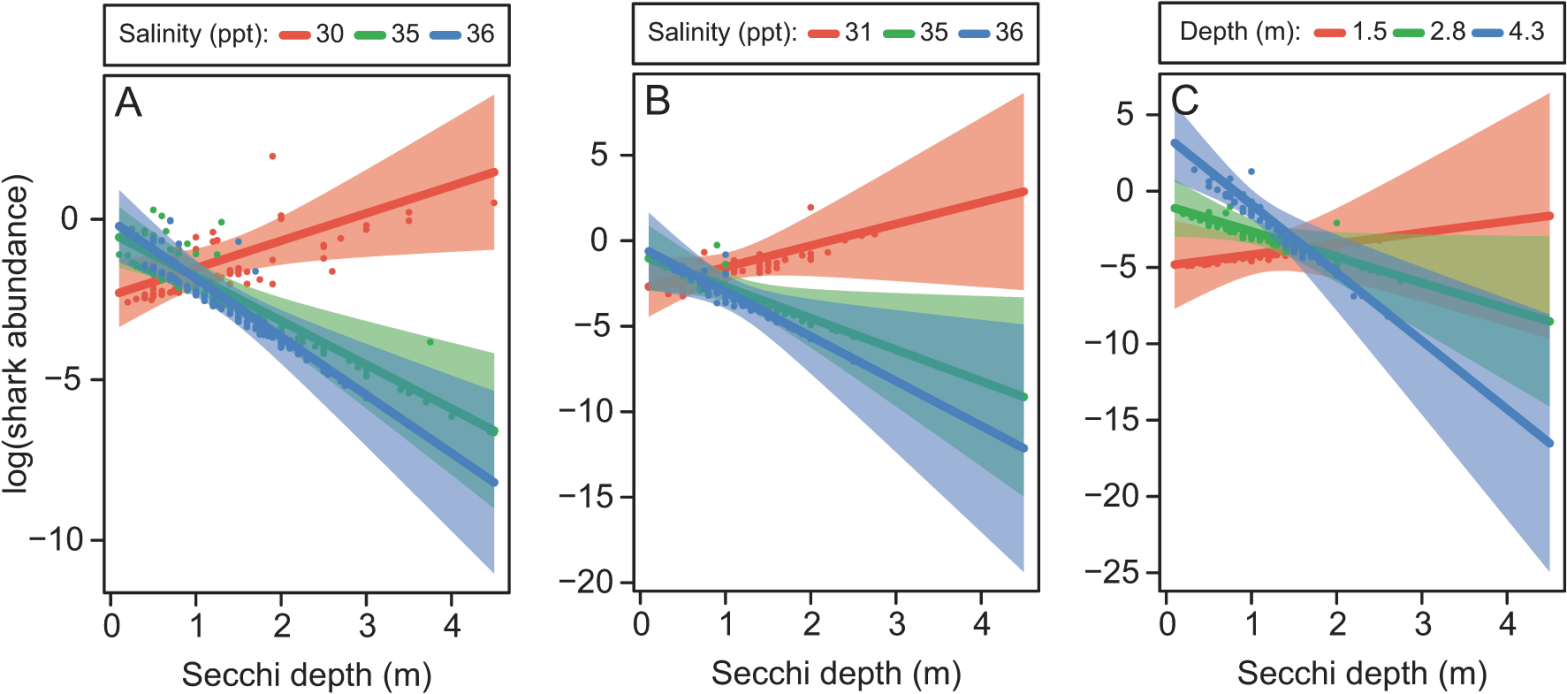
Modelled relationships between the abundance of immature pigeye sharks and highly influential variables in longline (A) and gill-net (B, C) samples. Shading represents 95% confidence intervals and points are partial residuals. Effects were plotted with additional variables held at their medians. To visualise interactions, cross-sections were taken at the 10^th^, 50^th^ and 90^th^ percentiles of the second variable of interest. The plotted longline model had dispersion statistic = 1.00 and negative binomial variance parameter *k* = 0.13. The plotted gill-net model had dispersion statistic = 0.84 and *k* = 0.11. Note that low values of secchi depth indicate high turbidity.

**Fig 6:**
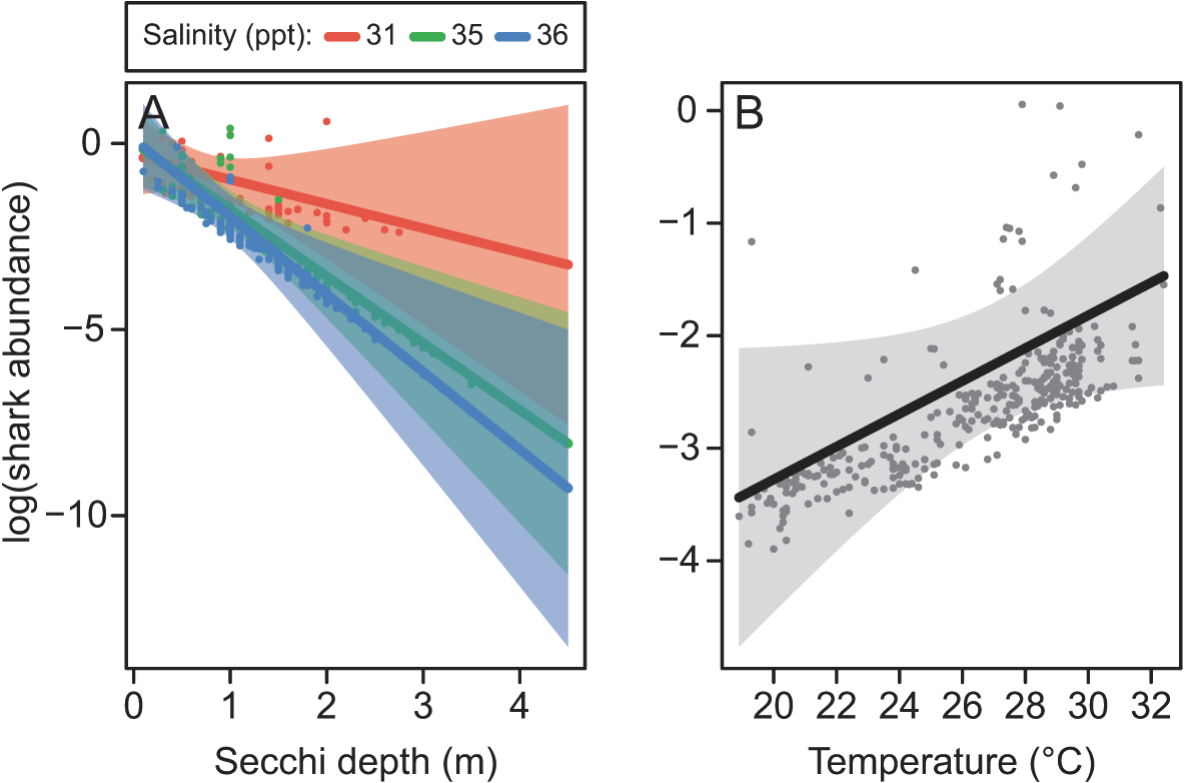
Modelled relationships between the abundance of immature scalloped hammerhead sharks in gill-nets and highly influential variables. Shading represents 95% confidence intervals and points are partial residuals. Effects were plotted with additional variables held at their medians. The influence of turbidity is plotted at the 10^th^, 50^th^ and 90^th^ percentiles of salinity. The model containing turbidity, salinity and temperature had dispersion statistic = 0.82 and negative binomial variance parameter *k* = 0.16. Note that low values of secchi depth indicate high turbidity.

Coefficients are on the linear (log) scale and so their effect is additive. Variables are listed according to mean RVI across species/sampling-method combinations. Asterisks denote variables that were not significant in model averaging but were significant (*P* < 0.05*; *P* < 0.0001**) in a single model containing only high-RVI variables. Although the coefficients for turbidity for pigeye and scalloped hammerhead sharks were positive, strong interaction with salinity or depth produced an overall negative relationship with decreasing turbidity (Fig. 5; Fig. 6).

#### Pigeye shark

A total of 68 and 44 pigeye sharks were captured using longlines and gill-nets, respectively. Of these, 63 and 41 immature individuals were included in longline and gill-net analyses, respectively. For both sampling methods, turbidity and its interaction with salinity were the most influential drivers of shark abundance (Table 3; Table 4). Abundance generally decreased with decreasing turbidity, however the opposite occurred at low salinities using both sampling methods (c. 30–31 ppt; Fig. 5A, B). For gill-nets, interaction between turbidity and depth suggested that relatively low-turbidity and shallow environments provided suitable habitat for young pigeye sharks (Fig. 5C). All high-order parameters were significant in model averaging (Table 4), and together explained 13% and 45% of deviance in pigeye shark abundance in longline and gill-net samples respectively. A negative relationship between pigeye shark abundance and distance from mangroves was also included in two of the three best-performing gill-net models (Table 3), however the RVI was relatively low (0.78), the model-averaged coefficient was non-significant (*Z* = 1.34, *P* = 0.18), and the coefficient in a single high-RVI model was weakly significant (*Z* = -2.17, *P* = 0.03). Therefore results on the influence of mangrove proximity on pigeye sharks were inconclusive.

#### Scalloped hammerhead shark

A total of 81 scalloped hammerhead sharks were captured in gill-nets and 73 immature individuals were included in the analysis. Scalloped hammerhead shark abundance decreased with decreasing turbidity, however this trend deteriorated at low salinities around 31 ppt (Fig. 6A). In addition, scalloped hammerhead shark abundance increased with temperature (Fig. 6B). Turbidity, salinity and temperature were present in all best-performing models (Table 3) and, together with interaction between turbidity and salinity, explained 29% of deviance in scalloped hammerhead shark abundance in gill-nets.

## Discussion

The relative abundance of immature sharks along an expanse of tropical coastline was related to environmental conditions. Of the 22 species sampled, blacktip, pigeye and scalloped hammerhead sharks were relatively abundant suggesting these species are important components of coastal ecosystems. Despite the overlapping distributions of these species, results indicated general and species-specific patterns in abundance which were characterised by a range of abiotic and biotic variables. In particular, relationships with turbidity were similar across species highlighting the importance of this variable in the functioning of coastal habitats and communal shark nurseries. Further, the influence of turbidity on the abundance of pigeye and scalloped hammerhead sharks varied similarly depending on salinity. Shark abundance and community structure have been found to vary along coastal stretches [40, 59]. In the present study, species-environment relationships, along with the demonstrated environmental heterogeneity between bays indicate that environmental variables are likely drivers of spatial variation in shark abundance and nursery function between bays.

The use of turbid coastal environments is considered to be an anti-predator strategy employed by young sharks [33, 60], although relatively few studies have investigated this relationship empirically. Turbid environments may also provide abundant prey for small sharks [61] or facilitate stealthy hunting strategies [33]. Immature blacktip, pigeye and scalloped hammerhead sharks were generally more abundant in turbid conditions, which aligns with previous findings for these species in northern Australia [62, 63] and in other locations [60, 64, 65]. In contrast, some small-bodied coastal species including spot-tail sharks *Carcharhinus sorrah* and slit-eye sharks *Loxodon macrorhinus* demonstrate a preference for relatively low turbidity, which may lead to reduced interspecific resource competition [63, 66]. There are also examples of turbidity having a modest [3] or negligible [67] effect on shark catch rates, potentially due to relatively uniform turbidity in some coastal waters (e.g. [3]). Therefore, the influence of turbidly appears to be species- and context-specific.

Salinity and temperature have important physiological implications for sharks [20, 68] and there are numerous examples of their influence on shark habitat use [3, 14, 36]. For example, the occurrence of immature scalloped hammerhead sharks in the north-eastern Gulf of Mexico increased with both salinity and temperature [15]. The utilisation of warmer water may represent behavioural thermoregulation [20] or may be related to seasonal fluctuations in the occurrence of this species. The influence of salinity on pigeye and scalloped hammerhead sharks was primarily related to its interaction with turbidity. The relatively high abundance in turbid water diminished at salinities often associated with coastal flood plumes (i.e. < 33 ppt; [41]). Previous acoustic tracking of immature pigeye sharks in northern Australia revealed that individuals moved away from freshwater sources during times of high freshwater input [53], which likely corresponded with increased turbidity and lower salinity. Similar movements in response to high river flows have been reported for juvenile rig *Mustelus lenticulatus* in a New Zealand estuary [69]. Stenohaline sharks typically inhabit a narrow range of salinities [11]. Therefore, immature pigeye and scalloped hammerhead sharks may have increased their use of relatively low-turbidity water to avoid low-salinity, albeit suitably turbid, conditions; thereby alleviating the metabolic costs of osmoregulation in salinities outside of their preferred range.

Sharks in shallow-water nurseries have been hypothesised to benefit from reduced predation risk because these depths can limit the access of large-bodied predators [30, 33, 70]. Contrary to this, the abundance of blacktip sharks in gill-nets increased with water depth suggesting that moderate depths up to 5 m also provide suitable habitat for young sharks in coastal environments. Immature common blacktip sharks were also shown to prefer depths around 5 m in the northern Gulf of Mexico [14, 15]. In contrast to blacktip sharks, acoustic tracking of pigeye sharks in north-eastern Australia revealed youngest individuals utilised depths around 2 m, although the depths occupied increased with age [62]. In the present study, although pigeye sharks were more abundant in turbid water, there was no clear influence of turbidity at depths around 1.5 m suggesting that shallow water alone may provide suitable refuge regardless of turbidity level. Therefore, the habitat use of young sharks is likely shaped by a multitude of direct, indirect and interacting relationships with their environment.

The results of this study showed blacktip sharks were more abundant in close proximity to mangroves. Because mangroves covered a large portion of coastline it was difficult to separate the influence of mangroves from that of distance from shore. However, other examples of positive associations between sharks and mangrove habitats corroborate the ecological relevance of this association. For example, elasmobranch abundance and number of species were higher in sites adjacent to mangroves in the eastern Indian Ocean [29] and young lemon sharks typically inhabit waters near or within mangroves [30]. The structural complexity of mangrove habitats may provide refuge for sharks within close proximity [28]. In addition, the high productivity of mangrove habitats can support large populations of teleosts and invertebrates on which young sharks feed [7]. However, high prey abundance does not necessarily increase prey availability for sharks. For example, the presence of prop roots or branches may impede successful hunting [71]. Alpheid burrows and seagrass are also thought to decrease the hunting success of young sharks [30, 72]. The present study indicated that mangroves may be important for young blacktip sharks, although the nature of this relationship remains poorly understood.

Similar species-environment relationships were observed between sampling methods, especially for pigeye sharks, providing support for the reliability of the results. For example, interaction between turbidity and salinity for pigeye sharks was apparent with both gears. However, some variation between gears emphasises the necessity to consider associated biases. For example, turbidity was less influential for blacktip sharks on longlines compared to gill-nets. Low turbidity may improve the ability of sharks to detect and avoid gill-nets [73] and thereby disproportionately affect gill-net efficacy. In addition, highly sensitive olfaction in sharks [74] may broaden the effective sampling range of baited longlines. The activity-specific nature of shark habitat use (e.g. feeding verses refuging; [25, 75]) may also dictate spatio-temporal variation in gear susceptibility. Similarly, Ulrich et al. [76] reported variation in the relative abundance and size composition of multiple species between gill-net and longline samples in coastal waters of South Carolina. Hence a combination of gears may provide a more-robust representation of shark abundance.

Investigations of species-habitat relationships are influenced by the spatio-temporal scales and variables considered, and the sampling and analysis methods used [15, 51, 52]. It was not possible to include all possible drivers of abundance in this study. Tides [77], river flows [53], DO [18], pH [17], substrate type [30], prey distribution [26], seagrass [29], coral reefs [78], and photoperiod [37] have also been related to the habitat use of sharks in coastal environments. Thus some or all of these variables may be important for immature blacktip, pigeye and scalloped hammerhead sharks along north-eastern Australia. Further, the correlative nature of our results leaves the underlying causative mechanisms unconfirmed. Nonetheless, by identifying the variables most strongly associated with shark abundances we provide a useful foundation for future studies.

Evidence that sharks respond to variations in environmental conditions coupled with significant spatial heterogeneity in these conditions between bays reveals them as probable drivers of spatial variation in habitat use between bays. Indeed, variable habitat use between bays is likely given stark variations in community structure and the relative abundance of individual species between inshore areas in this [40] and other locations [14, 15, 59]. For example, Rockingham Bay had the highest turbidity and highest abundance of immature scalloped hammerhead sharks [40]; whereas Edgecumbe Bay had the lowest turbidity and only one recorded scalloped hammerhead shark. Given that turbid water was identified as the strongest driver for this species, spatial variation in turbidity is a probable mechanism behind variations in abundance between bays. Similar patterns involving turbidity and the abundance of pigeye sharks were apparent between Repulse and Edgecumbe Bays. Spatial variation in the abundance of sharks has been demonstrated in other regions. For example, variations in the occurrence of common blacktip, bull and bonnethead *Sphyrna tiburo* sharks between Texas bays were attributed to variations in salinity, water temperature and proximity to tidal inlets [3]. Therefore, habitat diversity coupled with environmental preferences may drive intraspecific heterogeneity in shark nursery function across a region.

The habitat use of coastal sharks is particularly relevant in light of increasing anthropogenic impacts on coastal ecosystems such as fishing and coastal modification [reviewed in 12]. In addition, climatic events can cause extensive loss of seagrass and mangroves [79, 80]. The impacts of these disturbances on sharks remain poorly understood; although mangrove loss [81] pollution [82], thermal effluent [83, 84], and hydrodynamic changes [85] are reported to influence the habitat use or fitness of coastal sharks. Port capacity along north-eastern Australia is predicted to triple by 2020 [86], and this may exacerbate numerous pressures including benthos disturbance, hydrodynamic changes, pollutant introduction and remobilization, elevated suspended sediments, and noise pollution (reviewed in [87]). Given that this study identified turbidity, salinity and mangrove proximity as potential drivers of shark abundance, the aforementioned disturbances are likely to have direct effects on the occurrence and habitat use of the study species. Although multiple species were positively associated with turbid conditions, further research is needed to understand the ecosystem-level consequences of any perturbations to shark habitat use.

The use of environmentally heterogeneous landscapes provides the potential for portfolio effects which can mitigate the effects of environmental changes [88–90]. If young sharks in different bays are differentially impacted by environmental change or localised impacts, populations may benefit from enhanced resilience whereby the effects of disturbance in one area are buffered by production in others [89]. In addition, contributions from a diversity of habitats can reduce variability in the production of individuals across a region [59], which can in turn influence population growth [6]. Therefore the variable distributions of sharks observed here [40] may be an effective strategy to enhance population viability.
